# Modulation of HIV-1 replication in primary dendritic cells in contact with autologous CD4 T-lymphocytes

**DOI:** 10.1186/1742-4690-9-S2-P182

**Published:** 2012-09-13

**Authors:** B Su, ME Biedma, A Lederle, M Lambotin, C Moog

**Affiliations:** 1 INSERM U748, Université de Strasbourg, Strasbourg, France

## Background

Immature dendritic cells (DCs), which reside in genital mucosal surfaces, are among the first cells that encounter HIV-1. These cells poorly replicate R5-tropic HIV-1, but have been shown to efficiently transfer the virus to autologous CD4 T-lymphocytes. We have shown that HIV-1 replication was enhanced in primary HIV-1-loaded DCs when they are in close contact with autologous CD4 T-lymphocytes. Recently, SAMHD1 has been proposed to restrict HIV-1 replication in myeloid cells by decreasing deoxynucleoside triphosphate (dNTP) pools. We aimed to decipher whether SAMHD1 was involved in the increased HIV-1 replication observed in DCs coculture with CD4 T-lymphocytes.

## Methods

DCs were generated from human blood CD14^+^ monocytes. After 2 hours of incubation with HIV-1, DCs were added to autologous primary CD4 T-lymphocytes. The percentage of infection was quantified by flow cytometry (intracellular p24 staining). The expression of intracellular SAMHD1 was measured by flow cytometry and by western blot. Virus-like particles containing Vpx (VLP-Vpx) was used as control to decrease SAMHD1 expression.

## Results

HIV-1 replication was enhanced in DCs cocultured with autologous CD4 T-lymphocytes compared to DCs alone. Slightly lower levels of SAMHD1 expression were detected in infected cocultured DCs by flow cytometry. Moreover, addition of exogenous dNTPs to the culture significantly increased HIV-1 replication in DCs. As control, VLP-Vpx enhanced viral replication and decreased SAMHD1 expression in DCs.

## Conclusion

Preliminary results suggest that the stimulation of HIV-1 replication in DCs observed in coculture conditions was partially associated to the concomitant decrease of SAMHD1 expression in DCs. As HIV-1 replication was inhibited by Abs in DCs, further investigations are required to determine if SAMHD1 modulates HIV-1 replication in the presence of these Abs. Cross-links between the restriction factors and humoral immune response should be considered in the development of an effective anti-HIV-1 vaccine.

